# Impact of Certification of Competencies on Undergraduate Medical Students

**DOI:** 10.7759/cureus.77695

**Published:** 2025-01-20

**Authors:** Arvind K Yadav, Sakshi Singh, Meenu Pichholiya, Sangita Gupta

**Affiliations:** 1 Pharmacology, Geetanjali Medical College and Hospital, Udaipur, IND; 2 Pharmacology, American International Institute of Medical Sciences, Udaipur, IND

**Keywords:** certification, competency, feedback, indian medical graduate, pre post test

## Abstract

Background

A competency-based undergraduate curriculum reinforces the need for certification of certain essential skills. The development of competencies would be the main emphasis of teaching-learning and assessment, which would continue until the target competency was attained. This study was planned to evaluate the impact of certification of competencies among second-year students in the Department of Pharmacology in terms of knowledge and feedback.

Methodology

After receiving approval from the institutional ethics committee, this study was conducted on MBBS second-year students in the department of pharmacology at a tertiary care teaching hospital in Rajasthan, India. After teaching certifiable competencies in practical class, students were given standardized validated multiple choice questions as pre-tests before certification and post-tests after certification. The feedback was analyzed using a five-point Likert scale questionnaire to assess students’ satisfaction and attitude at the end of a session.

Results

A total of 123 second-year MBBS students who completed both pre- and post-tests were enrolled in the study. The mean score before and after the test was 10.90 ± 2.80 and 13.08 ± 2.90, respectively. There was a statistically significant difference between before and after test scores (p < 0.0001).

Conclusion

According to the study's findings, the certification of certifiable competencies showed a significant improvement in the performance of students after the intervention. This shows that it played an important part in improving students' knowledge. Thus, it is an effective tool to impart knowledge among students where discussions with faculty and feedback to students play important roles.

## Introduction

The Medical Council of India's "competency-based undergraduate curriculum," also known as competency-based medical education (CBME), will undoubtedly advance medical education and produce competent Indian Medical Graduates (IMGs) who can serve the community [1]. An outcome-based approach to the design, implementation, assessment, and evaluation of a medical education program using a structured framework of competencies is known as competency-based education [2].

The outcome-driven undergraduate curriculum has made a substantial effort to give the orientation and lifelong learning skills needed to provide good patient care. Early clinical exposure, electives, and long-term care are all part of the curriculum. The skill acquisition is a crucial aspect of the learning process in medicine. This CBME-based curriculum reinforces this aspect by requiring certification of certain essential skills. The most advanced degree of skill acquisition in the years before an internship is Show-How (SH) in a controlled or simulated environment. Few abilities necessitate independent performance and certification [1].

All competencies are taught in the practical class in detail with discussion. Certification is a process in which students are given feedback, and this process is repeated until the student is able to perform correctly. After the completion of the exercise to be certified, faculty members certify the exercises with one-on-one discussions and give feedback to students. Certification is a bidirectional process in which faculty also requires feedback from students about what can be improved in certification exercises. Feedback is a formal active engagement that is delivered with the intention of fostering the learner's development, improvement, and positive change through aided reflection on the task completed [3].

The certification process came into existence in India in 2019. In the literature search, no study was found that reported how students gain knowledge through certification, and feedback by students was also not reported after certification. Getting feedback from students will add a new perspective to our study. This study was planned to know the impact of certification of competencies on second-year undergraduate medical students.

## Materials and methods

Design and setting(s)

This educational interventional study was conducted in the Department of Pharmacology from 15/10/2022 to 30/04/2023 after ethical permission at a tertiary care teaching hospital in Rajasthan, India.

Participants and sampling

Professional MBBS students were included in the study. The students who refused to take part in the study were excluded.

Ethical considerations

This research was conducted only after receiving approval from the institutional ethics committee (Ref: GU.HREC.EC.2022.2.56). Informed consent was obtained from all the participants before the pre-test was given. The participation of the students in this study was completely voluntary and anonymous. Confidentiality of their personal information was maintained.

Data collection methods

A set of 20 questions from all the certifiable competencies to be certified were formed (Appendix 1). These questions related to competencies were discussed with experts in the Department of Pharmacology of different institutes, and appropriate modifications were made as per the suggestions given. This validated questionnaire was given to students as a pre-test and post-test for assessment of knowledge and awareness. Questions related to respective certifiable competency were also grouped together, and their mean pre-test and post-test scores were analyzed.

Students were taught about each certifiable competency in practical classes or small group teaching, and the duration of each practical teaching class was two hours (Appendix 2) [1]. They learned about competencies during the practical class in small groups. One faculty member was assigned to a group of 10 students. A set of questions was given to the students in class before starting the certification of competencies as a pretest. The defined number of procedures for each certifiable competency were done. In the classes conducted for certification, students were individually given one-on-one feedback by the faculty of the department about their performance. A total of eight to 10 faculty members were present during the certification process. The faculty evaluates the student’s exercise by reviewing their work, posing related questions, and giving feedback for a duration of five to seven minutes. Faculty establish the standards for the level of achievement or criteria that determine a satisfactory (meets expectations) or unsatisfactory (below expectations) grade (Appendix 3). If a required repeat exercise was given, the student had to do it again in the next class. Remedial training was given after a number of repeats. This process was continued until students get complete (C) score for each exercise in competency. After certifying all the certifiable competencies with a completed (C) score, post-test was given. In the end, feedback from the students was taken and analyzed using the Likert scale, where one stands for strongly disagreeing and five for strongly agreeing (Table 1).

**Table 1:** Feedback questionnaire items

	Questionnaire items
1	I was satisfied with the overall learning about certifiable competencies in the certification exercise.
2	I enjoyed learning in the certification exercise.
3	I was satisfied with the quality of my learning along with feedback during certification.
4	I was satisfied with the quantity of my learning along with feedback during certification.
5	Certification exercises stimulated my desire to acquire more knowledge through certifiable competencies.

Statistical analysis

Using Microsoft Office Excel 2010 (Microsoft® Corp., Redmond, WA), data were entered. Data were recorded in mean ± SD and number or percentage. Pre- and post-test data were compared using a paired t-test, and a p-value of less than 0.05 was regarded as significant.

## Results

One hundred twenty-three MBBS second-year students, out of a total of 250 students, were enrolled in the study and given both pre- and post-tests.

The mean score in the pre-test was 10.90 ± 2.80 (Table 2), with the lowest score being four and the highest score being 17 out of 20. The mean score after the test was 13.08 ± 2.90 (Table 2), with the lowest and highest scores being five and 19, respectively. There was a statistically significant difference between before and after test scores (p = 0.0001).

**Table 2:** Mean score of pre-test and post-test *Significant (by applying paired t-test)

Subjects	Pre-test (mean± SD)	Post-test (mean ± SD)	p-value
Students (n = 123)	10.90 ± 2.80	13.08 ± 2.90	0.0001*

The mean scores of respective competencies were also analyzed in both the pre-test and post-test. The mean score for P-drug competency in the pre-test and post-test was 0.57 ± 0.52 and 1.09 ± 0.72, respectively (p = 0.0001). The mean score for drug promotional literature (DPL) competency in pre-test and post-test was 3.07 ± 0.90 and 3.55 ± 0.86, respectively (p = 0.0001). The mean score for prescription writing in the pre-test and post-test were 4.38 ± 1.10 and 5.20 ± 1.156, respectively (p = 0.0108). The mean score for critical appraisal in pre-test and post-test was 2.88 ± 0.99 and 3.24 ± 0.98, respectively (p = 0.0047). A statistically significant improvement was found in each competency (Table 3).

**Table 3:** Pre-test and post-test scores of students in respective competency *Significant (by applying paired t-test)

Competency	Pre-test (mean ± SD)	Post-test (mean ± SD)	p-value
P-drug	0.57 ± 0.52	1.09 ± 0.72	0.0001*
Drug promotional literature	3.07 ± 0.90	3.55 ± 0.86	0.0001*
Prescription writing	4.38 ± 1.10	5.20 ± 1.15	0.0108*
Critical appraisal	2.88 ± 0.99	3.24 ± 0.98	0.0047*

**Figure 1:**
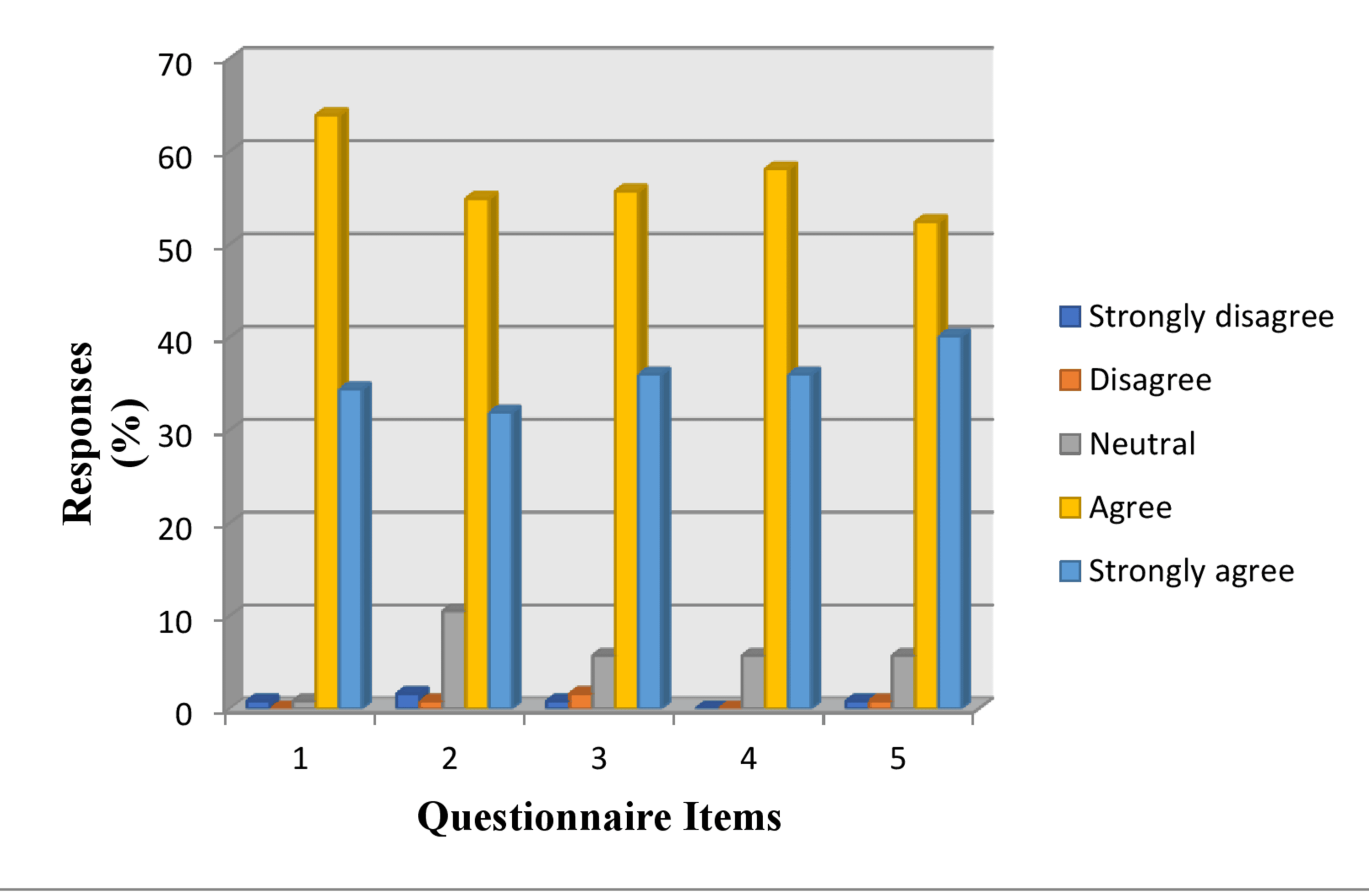
Feedback questionnaire analysis

Students' feedback questionnaire analysis showed that 63% of students agreed that they were satisfied with the overall learning about certifiable competencies in the certification exercise; 40% of students strongly agreed that certification exercises stimulated their desire to acquire more knowledge through certification competencies (Figure 1).

## Discussion

As a part of the new curriculum, certification of certifiable competencies is part of the assessment in all phases of the undergraduate teaching program. All three learning domains (cognitive, psychomotor, and affective) should be taken into consideration when creating an internal evaluation, and weightage should be given to these areas for assessment. The certification process fosters ownership of teaching-learning strategies and gives all teachers and students "hands-on" experience [2]. 

Pre- and post-test procedures are commonly used methods to assess the effectiveness of any educational program. This process gives feedback to the instructor by measuring the learner's starting knowledge level and the knowledge the learner acquired through the teaching. In this method, the student will be actively involved in education; the assessment is done more efficiently [4]. Multiple-choice questions are the most popular method for evaluating the cognitive ability of an individual. Thus, this study was planned to impart knowledge to the students using the certification process of the new curriculum.

In the present study, students were taught individual competency in their routine practical classes or small group teaching. Prior to certification, the mean pre-test score was 10.90 ± 2.80, in which the lowest score was four and the highest score was 17. These results showed that many students had also gained knowledge during routine classes. The mean post-test score after the certification process was 13.08 ± 2.90, where the lowest score was five and the highest score was 19. the minimum score was five, and the maximum score was 19. Certification is a process in which there is a one-on-one discussion of faculty with the students about that topic, and feedback is given to each student individually. Good feedback practice provides students with relevant information that will help them learn better and provide the teachers with the necessary information to improve their teaching experience [5]. Students get more chances to clear their doubts effectively with this method because the teacher gives attention to the only student who is allotted it. This implies that the certification process helped students improve their knowledge, which is seen by their improved mean post-test scores. It is also a good tool to improve the knowledge of low-scoring students as they get one-on-one attention and feedback from teachers. Other studies also reported similar results, which showed detailed feedback on individual tasks was revealed to be significant in terms of enhancing student performance [6,7]. Feedback should be a process of motivating the students in a positive way.

Most of the students (approximately 98%) were satisfied with the overall learning about certifiable competencies in the certification exercise, out of which 34% of them were highly satisfied. Certification helped students to learn better by understanding the topic in detail through one-on-one discussions with the faculty. More than 50% of students enjoyed the learning process during certification, as they were free to ask questions and resolve their doubts during this process.

More than 50% of students were satisfied with the quality of learning during certification. This could be because teachers gave proper time to each student and also taught them about their mistakes during this process, which improved the quality of learning. If students failed to complete the first attempt, then a repeat exercise was given to students in the next session. If they performed below expectation in repeat exercises and failed to understand the competency properly, a remedial session was conducted. In the remedial session, faculty taught them the same topic again, and a repeat exercise was given for the same. This process improved the overall quality of the certification process [8,9].

More than 50% of students were satisfied with the quantity of learning during certification. This could be because most of the certifiable competencies were related to their future clinical practice, which includes prescription writing, critical appraisal, DPL, and P-drug. The improvement of the health system's quality can be achieved by learning a good understanding and practical aspects of prescription writing, which in turn is capable of creating a more rational prescription [10-12]. Certification of competency in undergraduate coursework to write rational, accurate, and clear prescriptions for the specified condition will enhance their knowledge about prescription writing. Additionally, it will assist them in avoiding prescription-related mistakes in the future. In clinical practice, this will improve their ability to write prescriptions. Students showed statistical improvement after certification of prescription writing competency in the present study. A prescription audit is also an education exercise and, if carried out often, may help to improve the standard of care provided for patients by making a prescription more appropriate [13]. Rational use of drugs will be promoted by evaluating the prescription patterns according to WHO criteria [14,15]. Students showed statistical improvement after certification of critical appraisal competency in the present study. Certification of critical appraisal (audit) of given prescription will help students perform the exercises and discuss with faculty their mistakes. This will decrease the prevalence of prescribing errors and guide them to prescribe a rational prescription. The goal of the P-drug concept is to familiarize students with P-drugs that have been selected from the national essential drug list based on their safety, efficacy, suitability, and cost [16]. While it can be challenging to disregard the advice of seniors and specialists while choosing the P-drug, the students should be able to critically evaluate all of the available material and use it to choose their own P-drug [17,18]. Students showed statistical improvement after certification of P-drug competency in the present study. During certification exercises, students’ knowledge about how to select P-drugs based on different criteria is enhanced as they perform different exercises on their own. When students attempt exercises and receive help and encouragement from the teachers, they can improve their understanding of concepts that were difficult to grasp during practical class.

To spread the word about new medicines, DPL is employed as one of the marketing strategies. During the promotion of the literature, students should thoroughly consider the research findings and make their own judgments, as misleading and erroneous information is now regularly discovered in promotional literature [19,20]. Students showed statistical improvement after certification of DPL competency in the present study. Certification of different DPL exercises will guide them on how to judge different aspects of DPL practically. Every exercise is different, so they will be able to understand the concept in a better way. If they do not perform satisfactorily, they have to perform repeat exercises, which will again improve their understanding.

More than 60% of students agreed certification exercises stimulated their desire to acquire more knowledge through certifiable competencies. Students’ questions were answered more effectively, and they learned more from this process as each student got more personal attention with one-on-one learning. One-on-one teaching is more intense because it identifies the strong and weak points of a student [21]. Certification made an impact on students' performance and helped them gain more knowledge about the topic.

Limitations

This is the first of its kind study; no such study was conducted earlier. Thus, more studies are required to see the impact of certification as per the new CBME curriculum.

## Conclusions

According to the study's findings, the certification of certifiable competencies showed a significant improvement in the performance of students after the intervention. This shows that it played an important part in improving students’ knowledge. Thus, it is an effective tool for imparting knowledge among students, and one-on-one discussions with faculty and feedback to students play an important role. This type of formative assessment is where feedback is given to students for learning and also helps faculty to teach students in better and simpler ways in the future.

## Appendices

Appendix 1 

**Table 4:** Questionnaire for assessing certifiable competencies

Questionnaire									
Q1. Which of the following is the correct sequence of steps for the selection of P-drug?	Define the diagnosis, specify the therapeutic objectives, make a list of effective groups of drugs, and choose an effective group according to the criteria.	Define the diagnosis, make a list of effective groups of drugs, specify the therapeutic objectives, and choose an effective group according to the criteria.			Define the diagnosis, specify the therapeutic objectives, choose an effective group according to the criteria, and make a list of effective groups of drugs.				Specify the therapeutic objectives, define the diagnosis, make a list of effective groups of drugs, and choose an effective group according to the criteria.
Q2. What do you mean by P-drug?	Priority drug	Personal drug			Prerequisite drug				Personalized medicine
Q3. Is the following statement true/false?	Subscription includes doctor’s signature and registration number.								
Q4. Is the P-drug list the same everywhere?	Yes				No				
Q5. Is the following statement true/false?	Catchy words and claims are the same in drug promotional literature.								
Q6. Whether the following statement is true/false	Is handwriting part of rational prescribing?								
Q7. Which of the following is an essential list?	National List of Essential Medicines	WHO Model List of Essential Medicines			Hospital formulary				All of the above
Q8. Which of the following is a false statement about ℞?	Prayer to a Greek god	“Take thou of”			Derived from Chinese word				Prayer for healing of patient
Q9. What does “signatura” mean?	Direction to patient	Signature			Direction to pharmacist				Precaution
Q10. Which of the following is not included in the superscription?	Name of physician	Diagnosis			Specific instruction to patient				Qualification of doctor
Q11. Fill in the blanks. Reminder in drug promotional literature is considered after ______ years.									
Q12. Which of the following references should not be scrutinized?	Unpublished data		In press			Symposium proceedings		Indexed journals	
Q13. Which of the following is not included in rational prescribing?	Non-pharmacological instruction		Writing Latin abbreviation			Cost consideration		Drug should be written in capital letters	
Q14. Write full form of INN.									
Q15. Which schedule gives the name of the psychotropic drugs requiring special licenses for manufacture and sale?	Schedule Y			Schedule X			Schedule H		Schedule W
Q16. Is the following statement true/false?	Polypharmacy is when more than three medications are prescribed.								
Q17. Is the following statement true/false?	Drug promotional literature provides biased information.								
Q18. Write parts of inscription.									
Q19. Is the following statement true/false?	Physicians should rely on information provided by medical representatives.								
Q20. Is the following statement true/false?	Compounded prescription is the prescription in which drugs prescribed are supplied by the pharmaceutical companies in ready-prepared form by its non-proprietary or trade name.								

Appendix 2 

**Table 5:** List of certifiable competencies

Sr. no.	Competency no.	Competency	No. of procedures to be done independently for certification
1.	PH 3.1	Write a rational, correct, and legible generic prescription for a given condition and communicate the same to the patient.	5
2.	PH 3.2	Perform and interpret a critical appraisal(audit) of a given prescription.	3
3.	PH 3.3	Perform a critical evaluation of the drug promotional literature.	3
4.	PH 3.5	To prepare and explain a list of P-drugs for a given case/condition.	3

Appendix 3 

**Table 6:** Assessment of competencies

Competency addressed	Name of activity	Date completed (dd-mm-yy)	Attempt at activity: first or only (F), repeat (R), remedial (Re)	Rating: below (B) expectation, meets (M) expectation, exceeds (E) expectation	Decision of faculty: completed (C), repeat (R), remedial (Re)	Initial of faculty and date	Feedback received; initial of learner

## References

[1] Medical Council of India (2025). Competency Based Undergraduate Curriculum for the Indian Medical Graduate. Indian Medical.

[2] Medical Council of India (2025). Assessment Module for Undergraduate Medical Education. https://www.nmc.org.in/wp-content/uploads/2020/08/Module_Competence_based_02.09.2019.pdf..

[3] Medical Council of India (2019). Guidelines for Preparing Logbook for Undergraduate Medical Education Program. https://www.nmc.org.in/wp-content/uploads/2020/08/Logbook-Guidelines_17.01.2020.pdf..

[4] Shivaraju PT, Manu G, Vinaya M, Savkar MK (2017). Evaluating the effectiveness of pre- and post-test model of learning in a medical school. Natl J Physiol Pharm Pharmacol.

[5] Bashir MA, Kabir R, Rahman I (2016). The value and effectiveness of feedback in improving students’ learning and professionalizing teaching in higher education. J Educ Pract.

[6] Lipnevich A, Smith J (2008). Response to assessment feedback: The effects of grades, praise, and source of information. ETS Res Rep Ser.

[7] Mag AG (2019). The value of students’ feedback. 9th International Conference on Manufacturing Science and Education - MSE 2019 “Trends in New Industrial Revolution”.

[8] Modi SB, R BN, Kanakamma LG (2021). Effect of remedial teaching on academic performance of poorly performing students in pharmacology: a quasi experimental study. Int J Basic Clin Pharmacol.

[9] Gangadhar R, Veeraswamy C, Suseela SS (2019). Impact of remedial teaching for low achievers in pharmacology to improve their academic performance. J Clin Diagn Res.

[10] Gupta M, Tikoo D, Pal S (2020). Assessment of prescription writing skills and impact of an educational intervention on safe prescribing among the first-year postgraduate medical students of tertiary care hospital. AMEI’s Curr Trends Diagn Treat.

[11] Upadhyaya P, Seth V, Sharma M (2012). Prescribing knowledge in the light of undergraduate clinical pharmacology and therapeutics teaching in India: views of first-year postgraduate students. Adv Med Educ Pract.

[12] Singh S, Yadav AK, Pichholiya M, Kamlekar SK, Gupta S (2022). Evaluation of knowledge, attitude, and practice about the rational use of medicines among junior residents in a tertiary hare hospital in India. Pharmacol Clin Pharm Res.

[13] National Institute for Clinical Excellence (2025). Principles for Best Practice in Clinical Audit. oxford: Radcliffe Publishing.

[14] Joshi R, Medhi B, Prakash A (2022). Assessment of prescribing pattern of drugs and completeness of prescriptions as per the World Health Organization prescribing indicators in various Indian tertiary care centers: a multicentric study by Rational Use of Medicines Centers-Indian Council of Medical Research network under National Virtual Centre Clinical Pharmacology activity. Indian J Pharmacol.

[15] (2025). How to investigate drug use in health facilities: selected drug use indicators. https://www.who.int/publications/i/item/who-dap-93.1.

[16] Khilnani G (2008). The concept of personal drugs in the undergraduate pharmacology practical curriculum. Indian J Pharmacol.

[17] Singh NR (2008). P-drug concept and the undergraduate teaching. Indian J Pharmacol.

[18] Priyadarshini BG, Kumar RK (2017). A study on concept of P-drug selection among rural general practitioners. Int J Basic Clin Pharmacol.

[19] Rode SB, Salankar HV, Katole NT, Deshkar AT, Dadmal AA, Parate SV (2022). Critical appraisal of drug promotional literature in accordance with WHO guidelines. Cureus.

[20] Khakhkhar T, Mehta M, Sharma D (2013). Evaluation of drug promotional literatures using WHO guidelines. J Pharm Negative Results.

[21] Polloway EA, Cronin ME, Patton JR (1986). The efficacy of group versus one-to-one instruction: a review. Remedial Spec Educ.

